# Diastolic Murmurs

**Published:** 1904-05-28

**Authors:** 


### Hospital Clinics.

### DIASTOLIC MURMURS.

Dr. A. W. Hollis[1] mentions eight causes of diastolic murmur. They are:—Aortic and pulmonary insufficiency, persistent ductus arteriosus, aneurysms, mitral stenosis, cardio-respiratory influences, pericardial, and pleuro-pericardial friction.

1. *Aortic Insufficiency.*—In about 90 per cent. of the cases the quality of the murmur is almost constant, being blowing and diminuendo. It follows the first sound after a slight pause and occupies the rest of the diastolic period. In the other 10 per cent. of the cases it may be rough, musical, rumbling, crescendo, or of short duration, and may occupy any portion of the diastole. The intensity varies greatly; in most cases it is marked, but in slight cases, or when compensation is failing, it may be exceedingly difficult to detect. The exact site of the murmur is variable, and it may be heard anywhere over the cardiac area, but as a rule it has a point of maximum intensity and is transmitted towards the apex and to some extent upwards along the great vessels. Rarely it may be heard in the back, but only if the patient be thin and the murmur very loud. The points of maximum intensity, in order of frequency, are: (1) just to the left of the base of the ensiform cartilage; (2) over the second or third right interspace, near the sternum; (3) over the third left interspace near the sternum; and (4) at the apex. The explanation o th s variability is, Dr. Hollis states, that the point of maximum intensity represents the spot where the regurgitant stream impinges on the ventricular wall, and this spot is a variable one owing to differences in the condition of the affected valve. When the murmur is heard most loudly over the aortic area it must be due to vibration in the aortic ring itself.

The diastolic bruit which may sometimes be obtained in cases of aortic insufficiency by slightly compressing the great vessels with the stethoscope, Dr. Hollis points out, is difficult to obtain, is not constant, and may be met with where no organic valvular mischief is present. When a murmur is short and occupies an unusual period of the diastolic pause, some other cause than aortic incompetence should be suspected, such as aneurysm, or very high arterial pressure, or the murmur may be a cardio-respiratory one.

2. *Pulmonary Insufficiency.*—The murmur produced by lesion of the pulmonary valve is much more constant than that of aortic insufficiency. Its greatest intensity is always just to the left of the sternum in the second or third space, and unless intense it is not audible at the apex. It usually follows more quickly on the first sound than does the aortic regurgitant murmur, and is as a rule more harsh and rumbling than the latter.

3. *Persistent Ductus Arteriosus.*—This condition gives rise to a murmur which resembles that of pulmonary insufficiency; the condition may in some cases be distinguished from the latter by the presence of less cyanosis, less clubbing of the fingers, a small right ventricle, and an accentuated pulmonary second sound.

4. *Aneurysmal Diastolic Murmurs.*—These are of two kinds. The first and commonest is due to implication of the aortic valves and resembles that of ordinary aortic regurgitation. The second is rare and is produced by the forcing out of blood during diastole from an aneurysmal sac through its narrow opening. Other signs of aneurysm are necessary for a correct diagnosis.

5. *Mitral Stenosis.*—In this condition three varieties of diastolic murmur may be found. The most common and important is the presystolic. In this the maximun intensity is always at the apex and usually localised there. It is rough, rumbling, and crescendo, running into the first sound, which is short, high-pitched, and intense. The cause of this character of first sound is usually thought to be due to the small amount of blood in the left ventricle and the consequent ease with which it can empty itself, but Dr. Hollis attributes it to the coarse vibrations at the auriculo-ventricular septum stimulating the ventricle to a sudden contraction by irritation of the nervo-muscular mechanism of the heart. A presystolic murmur (Austin Flint) may be caused by aortic insufficiency, but in this case the ordinary diastolic murmur of this condition is nearly always present in addition. The second murmur due to mitral stenosis occupies most of the diastolic period. It is heard between the apex and the end of the sternum, and can only be differentiated from that of aortic incompetence by other signs. The third murmur in mitral stenosis is very short, soft, and blowing, and occupies the first part of diastole. It is due to the pent-up blood in the left auricle flowing swiftly into the left ventricle at the beginning of diastole; the ordinary presystolic is almost invariably present as well.

6. *Cardio-respiratory Murmurs.*—Diastolic as well as systolic murmurs may be produced in the lung tissue adjacent to the heart by the sudden change in size of that organ. They may be heard anywhere along the anatomical border of the heart, and if the breath is held the murmur is constant and uniform. Diagnosis may be very difficult, but the short duration of the murmur, its soft "puffy" character, together with the absence of organic signs, should make the case clear.

7. *Pericardial Murmurs.*—In pericarditis both systolic and diastolic murmurs are heard at some time during its course. The quality is superficial, rubbing, soft, or harsh. Pressure with the stethoscope may intensify the sound.

8. *Pleuro-pericardial.*—This murmur differs from the former in four particulars: the site is always at or a little above and internal to the apex; respiration has a decided influence on the sound; other signs of pericarditis are absent; indications of dry pleurisy are almost always present.

[1] Medical News, May 7.

